# Seq-ing Higher Ground: Functional Investigation of Adaptive Variation Associated With High-Altitude Adaptation

**DOI:** 10.3389/fgene.2020.00471

**Published:** 2020-05-14

**Authors:** James E. Hall, Elijah S. Lawrence, Tatum S. Simonson, Keolu Fox

**Affiliations:** ^1^Division of Pulmonary, Critical Care, and Sleep Medicine, Department of Medicine, School of Medicine, University of California, San Diego, La Jolla, CA, United States; ^2^Department of Anthropology and Global Health, University of California, San Diego, La Jolla, CA, United States

**Keywords:** genetic adaptation, genome editing, high-altitude adaptation, functional investigation, adaptive variation

## Abstract

Human populations at high altitude exhibit both unique physiological responses and strong genetic signatures of selection thought to compensate for the decreased availability of oxygen in each breath of air. With the increased availability of genomic information from Tibetans, Andeans, and Ethiopians, much progress has been made to elucidate genetic adaptations to chronic hypoxia that have occurred throughout hundreds of generations in these populations. In this perspectives piece, we discuss specific hypoxia-pathway variants that have been identified in high-altitude populations and methods for functional investigation, which may be used to determine the underlying causal factors that afford adaptation to high altitude.

## Introduction

Many of humankind’s smallest but greatest secrets are packaged within the human genome. Recent advancements have accelerated our ability to unravel these mysteries and discover how the genome contributes to shared and distinct variations in human traits, including those that have been essential for survival. Some of the most striking examples of adaptation within our species occurred in populations that migrated to the Tibetan, Andean, and Ethiopian highlands in the past several millennia. Physiologists first noted distinct characteristics among highland populations centuries ago, postulating that specific traits were helpful or harmful to challenges imposed by environmental hypoxia due to decreased oxygen availability at high altitudes (West, 1998). Given that many highland populations have persisted in such environments for hundreds of generations, it was hypothesized that genetic factors provided an adaptive advantage in these groups.

Within the past decade, it has become increasingly feasible to obtain insight into the evolutionary past of our species through genome-wide scans in search of adaptive signatures that highlight outlier patterns within the genome (Simonson, 2015). While many of the original studies of highland Tibetan, Andean, and Ethiopian populations were based on analysis of “tagging” single nucleotide changes scattered throughout the genome, whole genome sequencing (WGS) tools have afforded an opportunity to cultivate multiple large-scale genome datasets, which provide greater resolution for cross-population comparisons. Further technological and molecular advances have provided additional multi-omics insights (e.g., transcriptomics, epigenomics, proteomics, metabolomics), and gene-editing techniques are now available to manipulate and assess the functional consequences of specific genetic variants and their impact on molecular and physiological pathways. This wealth of information regarding genomic, -omics, and functional variation provides an opportunity to more comprehensively examine aspects of evolutionary change in highlanders and place this information into the context of other large-scale, publicly available data sets.

## Genomics and Association Studies

Bioinformatic screens of human genetic variation are increasingly used by geneticists and physiologists to better understand features of the human genome, including variable sites, chromosomal arrangements, and population-level variation. Genome-wide tests of selection have been successfully employed across continental populations, yielding candidate genes that are putatively associated with adaptation to high altitude (Beall et al., 2010; Bigham et al., 2010; Simonson et al., 2010; Yi et al., 2010; Alkorta-Aranburu et al., 2012; Scheinfeldt et al., 2012; Huerta-Sánchez et al., 2013). With regard to the specific challenge of hypoxia at high altitude, key players of the hypoxia inducible factor (HIF) pathway that sense and respond to changes in oxygen availability have been highlighted in hundreds of studies focused on genetic adaptation to high altitude (Bigham and Lee, 2014), and other non-HIF genes have also been reported in more than one study (Simonson, 2015). Given the HIF pathway has been implicated as a master transcriptional regulator of hypoxic response on an evolutionary time scale as far back as metazoans (Semenza, 1999; Giaccia et al., 2003; Wenger et al., 2005; Lendahl et al., 2009), these findings have been a major focus of high-altitude research. Last year, William Kaelin Jr, Sir Peter Ratcliffe, and Gregg Semenza were awarded the Nobel Prize in Physiology and Medicine for their ground-breaking research that provided the first insights into HIF signaling, highlighting the immense impact of this work on health and disease (Prabhakar, 2020).

Perhaps the most celebrated genes identified as targets of adaptation to high altitude are those involved in the HIF pathway, e.g., *EGLN1* and *EPAS1.* Both genes are among the top targets of adaptation in Himalayan populations (Beall et al., 2010; Bigham et al., 2010; Simonson et al., 2010; Yi et al., 2010), and *EGLN1* was identified among candidate genes initially described in Andeans as well (Bigham et al., 2009, 2010). *EPAS1* was recently detected in a test for early stage selection in Andean populations (Eichstaedt et al., 2017) and has been reported as a selection candidate gene in other highland populations in Central Asia (Pagani et al., 2012; Xing et al., 2013; Hackinger et al., 2016).

WGS analyses of DNA from Neanderthal and Denisovan genomes has also provided evidence that genetic material from these archaic human populations is present in modern humans today. Genetic variants in the *EPAS1* gene region in Tibetans, noted as one of the strongest adaptive signatures in Tibetans, is most similar to Denisovan DNA compared to DNA of other human populations (Huerta-Sánchez et al., 2014; Hu et al., 2017). While the functional variants have yet to be determined, these studies suggest archaic genetic admixture provided variation that helped Tibetans adapt to the high-altitude environment. This finding highlights the importance of understanding distinct population histories, and unique genetic backgrounds, in studies of genetic adaptation to high altitude.

## Phenotype Associations and Coding Variation

Despite tremendous progress on the genomics front, the precise variants that provide functional benefits for high-altitude adaptation remain largely unknown. Scientists are at the early stages of understanding which specific variants in and around the adaptive gene regions identified in highlanders provide functional benefits afforded by natural selection. Phenotype associations made thus far provide a means for prioritizing genes for further investigation. The first three genomic studies to examine relationships between putatively adaptive genes and phenotypes identified relationships between hemoglobin concentration and a combination of candidate genes that exhibited signals of selection, including *EGLN1* and *EPAS1* (Beall et al., 2010; Simonson et al., 2010; Yi et al., 2010). *EGLN1* encodes PHD2, one of three key prolyl hydroxylase oxygen sensors that target the α subunits of the HIF transcription factor for degradation under normoxic conditions, and *EPAS1* encodes HIF-2α, which serves to activate the expression of a few hundred genes in response to hypoxia. The downstream effects of these adaptive targets are extensive and relevant to multiple disease states, ranging from cardiopulmonary disease, metabolic dysfunction, to cancer (Semenza, 2020).

Two amino acid variations in PHD2 (*EGLN1*) were identified in Tibetans: p.Asp4Glu (p.D4E) and p.Cys127Ser (p.C127S) (Lorenzo et al., 2014). While these are located outside of the known functional domains (McDonough et al., 2006), effects on oxygen K_*m*_ and protein interactions have been observed, with different models for p.D4E/C127S mutations resulting in gain- or loss-of function (Lorenzo et al., 2014; Song et al., 2014). Due to the likely effects of these two mutations on protein-protein interactions and PHD2’s catalytic activity, further molecular characterization will be required to determine their specific role(s). The variants underlying adaptation at the *EGLN1* locus appear to be different in Andeans and remain to be determined (Bigham and Lee, 2014; Heinrich et al., 2019).

A single missense *EPAS1* variant p.H194R is reported as a target of selection in Argentinian highlanders (Eichstaedt et al., 2017), yet little is known about how this variation affects protein stability and protein-protein interactions with binding partners (e.g., ARNT and PHD2) in humans. However, several variants in non-human species adapted to altitude have been identified and modeled as gain- (Song et al., 2016; Liu et al., 2019) and loss- (Gou et al., 2014) of-function. Given the highly conserved nature of *EPAS1* among species, each of these variants provide critical insight into the convergent adaptation to life at high altitude.

While these proteins are involved in hypoxic signaling, many others are also involved, making it difficult to discern the effects of single mutations in a complex signaling network. Reverse engineering these precise mutations, whereby site-specific point mutations are introduced into cells of a standard background in controlled environments, provides an opportunity to functionally investigate impacts on protein structure, protein-protein interactions, and signal transduction as well as gene expression and downstream effects due to alterations in regulatory variants. Experiments in an “adapted” genome are also valuable if multiple genetic targets of selection in that background are carefully considered.

## Genome Editing and Functional Investigation of Adaptation

While WGS and association studies are great tools for identifying and prioritizing genetic variants that might be involved in both natural selection and disease phenotypes, these correlative findings do not provide information regarding causality. Gene-editing tools offer physiology and population genetics communities opportunities to investigate the roles of candidate variants and identify precise sites underlying adaptation to hypoxia inherent to high altitude.

In addition to using existing tools, which allow for the reverse engineering of individual single nucleotide variants (SNVs) in isogenic cell lines, multiple SNVs may be examined simultaneously (i.e., multiplex introduction of population specific haplotypes) (Hoehe et al., 2019). This new approach allows for functional investigation of multiple epistatic mutations that are under selection, even if they are located on different chromosomes. These tools could be used to endogenously introduce population-specific high-altitude-adapted haplotypes in physiologically relevant cells lines that recapitulate hypoxic signaling, oxygen metabolism, and posttranslational modification of hypoxia-inducible transcription factors (e.g., A549 Human Lung Adenocarcinoma cells, Sw.71 human trophoblast cells, and ASC52 adipose derived mesenchymal stem cells).

Other publicly available tools may be used to assist in understanding the relationship between genetic variation and tissue-specific expression, including the Genotype-Tissue Expression (GTEx) database (based on data from 17,000 samples, representing 54 tissues from 948 different donors). This resource, in tandem with epigenetic information gathered from resources such as The Encyclopedia of DNA Elements (ENCODE) Consortium, provide the potential to uncover complex gene regulation networks within a wide array of human tissues (Bernstein et al., 2010; Dunham et al., 2012). Such information may be applied to better understand data from various human studies, including those at high altitude, where tissue- and cell-specific sampling is limited. A brief table of techniques and tools for investigating genetic variants in this context are provided in Table 1.

**TABLE 1:** Techniques and tools to investigate genetic variation and their applications.

	**Technique or tool**	**Pros**	**Cons**
Techniques to introduce genetic variants	Single-base editing (Cas9)	• Introduces specific nucleotide variant within the genome • High efficiency	• Chance of bystander mutations
	Over-expression of protein variants	• Introduce protein variants into cellular systems	• Potential imbalance in systems regulated
		• Options for controlled induction	• Expressed proteins may end up in inclusion bodies
			• Production is slow in stable cell lines, especially in the case of selective cloning
	Non-homologous end joining (NHEJ); Homology-directed repair (HDR)	• Used to edit within non-coding regions as compared to overexpression	• Programmable nucleases can have varying success rates • Random insertions/deletions (indels) • Higher rates of mutation
Techniques to assay gene expression	RNA interference (RNAi)	• Targeted knockdown of expressed transcripts	• Variability and incompleteness of knockdowns • Purely a loss-of-function technique • Unmodified siRNA easily degraded • High-turnover transcripts hard to silence
	RNA-seq	• Genome-wide insight into transcript expression (protein-coding and non-coding genes, microRNAs) • Capacity to explore unannotated species • Good option if total RNA is low (1 ng–2 μg)	• Files may be many gigabytes; expense of procedure and data storage • Sensitive to library preparation protocol
	Expression Microarray	• More affordable • Data files typically only a few MB	• Limited to available probes • High background noise • Limited dynamic range
Techniques to assay proteomics and metabolomics	Proteomics	• Identification of multiple proteins within a biological sample • Used to characterize splice variants and post-translational modifications • Quantitative applications available for determining differences in protein levels	• Loss of intact protein information • Multiple post-translational modifications, chemical byproducts, and unintended cleavages can make it difficult to identify protein fragments in a sample • Multiple proteins may share similar sequences making it difficult to assign fragment origin • Quantitative applications rely heavily on accuracy, precision, repeatability and specificity; Complex samples may have to be further refined and run multiple times
	Metabolomics (Liquid chromatography-mass spectrometry LCMS; Gas chromatography-tandem mass spectrometry GC-MS)	• Identification of peptides and other small molecules • Quantitative applications available for determining differences in molecular levels • High sensitivity • Many databases available for identifying spectra	• High number of spectral signals can require time and expertise for proper identification • Quantitative applications rely heavily on accuracy, precision, repeatability and specificity • Complex samples may have to be further refined and run multiple times
Examples of on-line tools used to examine genetic variants	UCSC Genome Browser https://genome.ucsc.edu	• Provides genome assemblies and annotations from vertebrates and model organisms with tools for viewing and accessing data • Provides detailed tracks with information from various platforms and studies (ENCODE, GTEx, etc.)	
	Gene Cards https://www.genecards.org	• Helpful information regarding gene pathways, regulation, tissue expression, phenotype, and genome-wide association studies data	–
	Ensembel Genome Browser https://uswest.ensembl.org/index.html	• Customizable • Best for single nucleotide variant (SNV) analysis	–
	Genome Data Viewer https://www.ncbi.nlm.nih.gov/genome/gdv/	• Provides information on a large number of species (∼100) • Includes genetic and cytogenetic data • Ability to view different assemblies side-by-side • Offers variety of coordinate systems (i.e., cM, cRay, basepairs) • Useful for big-picture analysis	–

## Future Perspectives on Functional Investigation

**FIGURE 1:**
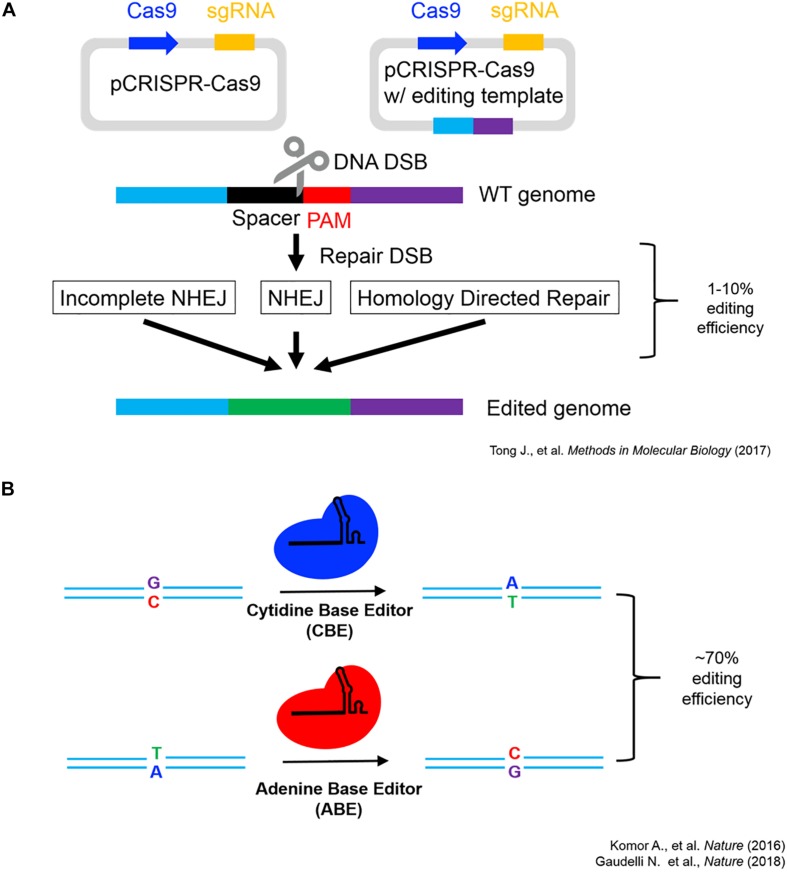
CRISPR-Cas9 schematics and editing efficiency. **(A)** CRISPR-Cas9 genome editing working model modified from Tong et al. (2018) showcasing the two major routes genome editing via DNA double-strand break repair utilizing non-homologous end joining (NHEJ) or homology-directed repair (HDR) (Tong et al., 2018). **(B)** Single-base editing utilizing modified Cas9 fusion proteins (Komor et al., 2016; Gaudelli et al., 2018).

As seen in Figure 1, the endogenous introduction of putatively adaptive alleles using a precision genome editing (i.e., base-editing signatures of natural selection into isogenic cell lines) offers an effective proof-of-concept for the functional investigation of locally adapted SNVs (Komor et al., 2016; Gaudelli et al., 2018; Tong et al., 2018). However, reverse engineering individual SNVs associated with high-altitude adaptation does not represent the functional investigation of the full spectrum of human genetic variation (i.e., haplotypes, structural variation (SV), and epigenetic inheritance, etc.). Such heterogeneity can be functionally investigated using a number of techniques, and multiple avenues for growth are anticipated to investigate signatures of natural selection in both *in vivo* and *in vitro* model systems. The development of multiplexed base-editing systems, which introduce point mutations in aggregate (i.e., introduce haplotypes under selection), will allow for functional investigation of not only epistatic interactions under selection, but also the functional impact of the inheritance of entire haplotype blocks in model systems (Hoehe et al., 2019).

### Structural Variation

Assessments of structural variation (SV), also known as copy number variation (CNVs), include identification of insertions, deletions, inversions, and translocations that may help explain some unaccounted heritability (Girirajan and Eichler, 2010). Early analyses from SNV-based microarrays identified a 3.4 kb CNV (deletion) ∼80 kb downstream from *EPAS1* in Tibetans that is found at low frequency in other populations (Lou et al., 2015). Newly developed population-specific genome assemblies (Hsieh et al., 2019) allow for the identification of novel SVs as well as the discovery of previously unknown human genes. However, with exception of a recent long-read *de novo* assembly of a Tibetan individual, population-specific SV has been largely unexplored with respect to high-altitude adaptation (Ouzhuluobu et al., 2019). Ouzhuluobu et al. (2019) identified a 163-bp deletion within an intergenic region of the *MKL1* gene (megakaryoblastic leukemia translocation 1), which has been associated with lower systolic pulmonary arterial pressure in Tibetans (Ouzhuluobu et al., 2019).

High-resolution population-specific *de novo* assemblies may also be compared to archaic hominid assemblies (e.g., Neanderthal and Denisovan) to illuminate population-specific evolutionary histories. Recent comparisons of high-altitude adapted populations from the Himalayas and archaic hominids suggest a higher frequency of genome-wide archaic hominid introgression in Himalayan populations relative to East Asian genomes (Huerta-Sánchez et al., 2014). In the *de novo* assembly study, a potentially introgressed archaic-hominid sequence (i.e., 662-bp intronic insertion) was identified in the *SCUBE2* gene, which has been linked with lung function (i.e., FEV1/FVC ratio) in Tibetans (He et al., 2019; Ouzhuluobu et al., 2019). Many high-altitude adapted SVs will require functional investigation in the appropriate *in vitro*/*vivo* systems as new genome-editing tools emerge.

### Epigenetics and Transgenerational Inheritance

Nuclear genome heterogeneity does not explain the full spectrum of high-altitude adapted phenotypes. Characterizing environmental exposure (past or present) has the potential to offer key insights into epigenetic and transgenerational inheritance. Previous research indicates that altitude-induced epigenetic changes occur (Childebayeva et al., 2019), and DNA methylation in the promoter region of *EPAS1* was lower in individuals living at high versus low altitude in Peru (Childebayeva et al., 2019). Another key factor is the number of years lived at high altitude (i.e., there is a positive association within a known methylated locus dependent on the number of years subsisting at high altitude). Overall, these data suggest that epigenetic modifications are potentially involved in high-altitude adaptation both at birth and over the course of a lifetime (Julian, 2019).

Many genomic studies suggest regulatory variation underlies responses to hypoxia (Frisancho, 2009; Nanduri et al., 2012, 2017; Hartley et al., 2013; Brown and Rupert, 2014; Azad et al., 2017; Hu et al., 2017). Multiple studies in other fields have used next-generation sequencing to reveal genome-wide associations between epigenetic modifications and transcriptional regulatory elements and chromatin accessibility (Fukushima et al., 2019). However, there is a dearth of research regarding such data at altitude. Nonetheless, we anticipate the reverse engineering of specific epigenetic modifications and transcriptional states associated with high-altitude adaptation using targeted *in vivo*/*in vitro* epigenome editing systems will provide an opportunity to tease apart direct causal/mechanistic relationships.

### Organoids and Assembloids

While isogenic cellular models offer a relevant system to functionally evaluate mutations that are putatively under selection, two dimensional (2D) *in vitro* cellular systems have a number of limitations. Three dimensional (3D) cellular models (i.e., tissue-specific organoids) offer the opportunity to functionally investigate the effects of cellular development, self-organization, and tissue-specific, organ-specific function (Sterneckert et al., 2014; Hubert et al., 2016; McCauley and Wells, 2017). 3D cellular organoids are collections or aggregations of organ-specific cells developed from pluripotent stem cells or organ progenitor cells. The cellular aggregates are self-organizing and may be used to determine spatially restricted lineage commitment similar to *in vivo* cellular models. When differentiated, 3D organoids are capable of recapitulating some of the functions of an organ of physiological interest (e.g., contraction, neural activity, endocrine secretion, filtration, excretion, etc.).

Precision genome-editing systems could be used to introduce high-altitude specific mutations (e.g., the SNVs reported in *EPAS1* or *EGLN1*) in pluripotent stem cell tissue systems that may be differentiation into physiologically relevant organoid systems. For example, human blood vessel organoids engineered as models of diabetic vasculopathy could also be genome-edited to create isogenic cellular models to functionally investigate the role of specific mutations in the development and arborization of vascular structures/networks putatively implicated in high-altitude adaptation (Wimmer et al., 2019; Wörsdörfer et al., 2019). Utilization of genome-edited 3D organoid models is not limited to vascular structures (For example, through recent emergence of dynamic “assembloid” systems, investigators are able to engineer and self-assemble multiple compartmentalized brain regions from human pluripotent stem cells and asses their connectivity (Trujillo and Muotri, 2018; Yoon et al., 2019). These technological advancements not only allow for the functional investigation of layered tissue-specific development but the functional investigation of the interplay between sub-tissue systems. They offer a glimpse into the future of functional investigation of high-altitude adapted populations, their physiology, and the dynamic relationships between organs and physiology under conditions of hypoxia.

### Single-Cell Genome Sequencing

Another avenue for growth regarding the functional investigation of high-altitude adaptation is the emergence of single-cell DNA and RNA analyses. Single-cell genome sequencing analyses allow for analysis of the entire transcriptome that may be integrated with genome, proteome, and metabolome information. By reverse engineering SNVs *via* precision genome-editing systems into the appropriate physiological cellular model, it is possible to provide complementary high-resolution views of -omics pathways with in single cells adapted to hypoxia.

### Reverse Engineering Hypoxic Environments

While physiologically relevant isogenic cell lines combined with genome-editing technology offer great insight into some of the biological mechanisms involved in high-altitude adaptive phenotypes, we recognize that evaluations of function and mechanism require consideration of environmental pressure(s) at high altitude (Fox et al., 2020). During hypoxia, HIF transcription factors are stabilized and regulate various genes such as those involved in oxygen transport, and there are multiple methods used in laboratory settings to induce hypoxia in physiologically relevant cell cultures (Wu and Yotnda, 2011). We anticipate researchers will introduce putatively adapted mutations in physiologically relevant cell lines to simulate conditions of tissue-appropriate levels of hypoxia in control incubation environments. Other species that evolved to subsist at high altitude may be used to identify mutations associated with hypoxia (Storz and Scott, 2019), and these mutations could be edited in the embryotic state to either remove or introduce mutations associated with hypoxia adaptation.

## Seeking Higher Ground With Indigenous Peoples

The potential to engineer new tools to functionally investigate genetic variation is an exciting prospect as it establishes greater accountability in research (e.g., in population genetics, reducing the tendency toward evolutionary adoptionism narratives (Gould et al., 1979). While some researchers overlook the importance of oral history, ethnography, linguistic, and archeological data when drawing conclusions about phenotypic observations and evolutionary analyses (e.g., GWAS and polygenic risk scores) (Nielsen, 2009), it is important to recognize that Indigenous populations should be included in the co-development of evolutionary and medically actionable narratives surrounding human genetic variation collected in their community (Jackson et al., 2019; Fox et al., 2020). Additionally, echoing previous observations by Lewinton and Gould, both ‘empirical evidence and detailed theoretical considerations’ should be used for evolutionary explanations of phenotypic variation observed in various Indigenous populations (Claw et al., 2018).

With the generation of data presented thus far and the ability to manipulate and test functional relevance, the scientific community will continue to gain meaningful insights into hypoxia adaptation. The mechanistic characterization of these observations, through the identification of adaptive gene targets like *EGLN1* and *EPAS1*, would not be made possible without partnerships in Indigenous communities thriving at high altitude. Understanding the function of specific variants in these groups contributes to a more complete picture of the human story and some of the most notable examples of adaptation within our species.

## Author Contributions

JH, KF, and TS designed the article. JH and KF designed the figures. JH, EL, TS, and KF drafted and contributed comments to the final manuscript.

## Conflict of Interest

The authors declare that the research was conducted in the absence of any commercial or financial relationships that could be construed as a potential conflict of interest.
